# Supplementary material for: Accurate Recapitulation of Chikungunya Virus Complete Coding Sequence Phylogeny Using Variable Genome Regions for Genomic Surveillance
Source: Viruses. 2024 Jun 7;16(6):926. doi: 10.3390/v16060926 (PMC11209212; doi:10.3390/v16060926)
Supplement: Supplementary file 1 [file viruses-16-00926-s001.zip › viruses-3019230-supplementary.pdf]

Table S1. Detailed sequence data from the six alignments used to assess the sensitivity of various regions in discriminating between sequences from different lineages and origins.

Alignment 1			Alignment 2			Alignment 3		
**Accesion**	**Year of isolation**	**Country**	**Accesion**	**Year of isolation**	**Country**	**Accesion**	**Year of isolation**	**Country**
LC259082	1958	Thailand	KY703888	2015	Nicaragua	KY703945	2015	Nicaragua
LC259092	2016	Cuba	KY703894	2015	Nicaragua	KY703946	2015	Nicaragua
MN462644	2015	Ecuador	MF580946	NA	NA	MH754507	2017	Italy
MN462652	2015	Ecuador	MF774613	2016	Pakistan	KX262986	1983	Senegal
LC500218	2015	Aruba	MF774615	2016	Pakistan	KX262988	1988	Thailand
MN462653	2015	Ecuador	MG049915	2017	Italy	KX262987	1996	Thailand
MN462654	2015	Ecuador	MG280943	NA	NA	MH823664	2017	Brazil
MN462655	2015	Ecuador	MG649971	NA	NA	KX262990	1959	NA
MN462658	2015	Ecuador	KY703905	2015	Nicaragua	MH823665	2017	Brazil
MN462659	2015	Ecuador	MG649978	NA	NA	KX262995	1983	Senegal
KJ679577	2011	India	MG649980	NA	NA	AB860301	2013	Philippines
MN630017	2019	Australia	MG649982	NA	NA	MK993755	2016	Brazil
MN756625	2019	China	KT308159	2012	Philippines	KY703961	2015	Nicaragua
MN974203	2019	Thailand	KT308160	2012	Philippines	KY703962	2015	Nicaragua
MN974204	2019	Thailand	KY703911	2015	Nicaragua	KY038946	1975	CAR
MN974205	2019	Thailand	EF027139	NA	India	KY038947	1983	CAR
MN974206	2019	Thailand	EF027140	NA	India	KY057363	2016	India
MN974208	2019	Thailand	EF027141	NA	India	MK120194	2017	Italy
MN974211	2018	Thailand	MG912993	2017	China	MK120196	2017	Italy
MN974213	2019	Thailand	MG921596	2015	Mexico	MK120198	2017	Italy
MN974216	2018	Thailand	EF452493	NA	Thailand	HM045784	1984	CAR
MN974217	2018	Thailand	EF452494	NA	USA	HM045785	1966	Senegal
MN974218	2018	Thailand	MH000705	2016	Brazil	HM045786	1964	Nigeria
MN974219	2018	Thailand	MH124581	2016	India	HM045787	1995	Thailand
MN974222	2018	Thailand	MH400249	2017	China	HM045788	1973	India
MN974223	2018	Thailand	MH423797	2016	Kenya	HM045789	1988	Thailand
AY726732	NA	Senegal	MH423799	2016	Kenya	HM045790	1985	Philippines
MK551553	2016	India	MH423803	2016	Kenya	HM045791	1983	Indonesia
JC739988	NA	NA	MH507158	2017	Italy	HM045792	1956	South Africa
MT526800	2017	Kenya	MH647208	2017	Singapore	HM045793	NA	CAR

Alignment 4			Alignment 5			Alignment 6		
**Accesion**	**Year of isolation**	**Country**	**Accesion**	**Year of isolation**	**Country**	**Accesion**	**Year of isolation**	**Country**
HM045795	1976	South Africa	KY703979	2015	Nicaragua	OL898681	2021	Brazil
HM045796	1995	Thailand	MK473623	2016	India	OL898683	2021	Brazil
HM045797	1985	Indonesia	MK473626	2016	India	OL898687	2021	Brazil
HM045798	1966	Senegal	MK473627	2016	India	OL898688	2021	Brazil
HM045800	1985	Philippines	MK473628	2016	India	OL898690	2021	Brazil
HM045802	NA	Thailand	MK473634	2016	India	OL898693	2021	Brazil
HM045803	1963	India	MT668625	2019	China	OL898694	2021	Brazil
HM045804	NA	Senegal	MK518395	2017	Brazil	OL898701	2021	Brazil
HM045805	1976	South Africa	MW110477	2019	China	OL898702	2021	Brazil
HM045806	1986	India	MW110476	2019	China	OL898704	2021	Brazil
HM045807	1965	Nigeria	MW110474	2019	China	OL898705	2021	Brazil
HM045808	1978	Thailand	MT640256	2020	Thailand	OL898706	2021	Brazil
HM045809	1960	DRC	MT495607	2019	Thailand	OL898707	2021	Brazil
HM045810	1958	Thailand	MT495606	2019	Thailand	OL898709	2021	Brazil
HM045811	1953	Tanzania	MT495605	2019	Thailand	OL898710	2021	Brazil
HM045812	1982	Uganda	MW042254	2014	India	OL898698	2021	Brazil
HM045813	1963	India	MT526902	2017	Brazil	OL898712	2021	Brazil
HM045814	1975	Thailand	KY703997	2015	Nicaragua	OL898713	2021	Brazil
HM045815	1979	Senegal	MW473668	2018	USA	OL898699	2021	Brazil
HM045816	1966	Senegal	MW248364	2019	China	OL898715	2021	Brazil
HM045817	2005	Senegal	ON009843	1953	Tanzania	OK316990	2019	China
HM045818	1981	NA	OL898666	2021	Brazil	OK316991	2019	China
HM045819	1993	Senegal	OL898667	2021	Brazil	OK316992	2019	China
KY703974	2015	Nicaragua	OL898668	2021	Brazil	OK316993	2019	China
HM045820	1993	NA	OL898670	2021	Brazil	KY751908	2016	Australia
HM045821	1963	Senegal	OL898671	2021	Brazil	L37661	NA	USA
HM045822	1978	CAR	OL898674	2021	Brazil	HQ846359	2010	China
HM045823	1962	Angola	OL898675	2020	Brazil	KJ451622	2013	Micronesia
MK468620	2017	Bangladesh	OL898676	2021	Brazil	MN402886	2019	China
MK468801	2018	Thailand	OL898677	2021	Brazil	AF369024	NA	NA

Table S2. Detailed sequence data employed to evaluate the suitability of high variable genome regions of CHIKV for outbreak tracking through genomic surveillance.

Accession	Year of isolation	Country	Genotype
AB860301	2013	Philippines	Asian and Caribbean
FJ807897	2007	Indonesia	Asian and Caribbean
KF318729	2012	China	Asian and Caribbean
KJ451623	2013	Micronesia	Asian and Caribbean
KP164567	2014	Brazil	Asian and Caribbean
KP164572	2014	Brazil	Asian and Caribbean
KR046232	2014	Trinidad and Tobago	Asian and Caribbean
KR559472	2014	El Salvador	Asian and Caribbean
KR559478	2014	Haiti	Asian and Caribbean
KR559480	2014	British virgin islands	Asian and Caribbean
KR559488	2014	Honduras	Asian and Caribbean
KR559491	2014	Colombia	Asian and Caribbean
KR559493	2014	American Samoa	Asian and Caribbean
KR559496	2014	Guyana	Asian and Caribbean
KT308159	2012	Philippines	Asian and Caribbean
KT308160	2012	Philippines	Asian and Caribbean
KT308161	2012	Philippines	Asian and Caribbean
KT308162	2012	Philippines	Asian and Caribbean
KT327163	2014	Mexico	Asian and Caribbean
KT327167	2014	Mexico	Asian and Caribbean
LN898093	2013	Martinique	Asian and Caribbean
LN898098	2014	Guadeloupe	Asian and Caribbean
MK468801	2018	Thailand	ECSA
OL898702	2021	Brazil	ECSA
MK468620	2017	Bangladesh	ECSA
MW248364	2019	China	ECSA
HM045785	1966	Senegal	West African
HM045807	1965	Nigeria	West African
HM045804	2010	Senegal	West African
AY726732	2005	Senegal	West African

Table S3 supplement. PCR conditions and primers proposed for NSP1, NSP3 and E2 partial gene amplification.

Reaction Mix

Reagents	Volumen
**RNA**	5 µl
**One step Buffer 2x**	10 µl
**Primer Mix**	2 µl
**Enzyme Mix**	0.2 µl
**PCR grade water**	2. 8 µl
**Total**	20 µl

Thermal cycler program

- 42°C by 60 min.
- 94°C by 3 min.
- 35 cycles:
  - 94 ° C by 30 seg.
  - 60 ° C by 30 seg.
  - 68 ° C by 45 seg.
- 68 ° C by 5 min.
- Hold 4 ° C

Primer	Sequence
Chik_NSP1 Forward	5'- ACTATCTGCTTCCCGTGGTC-3'
Chik_NSP1 Reverse	5'- TACGCTTCGACCGCATACCT-3'
Chik_NSP3 Forward	5'- TCGATCAGGCAGAAATGCCC-3'
Chik_NSP3 Reverse	5'- CTTGGTGCTCCGAAGGAGAT-3'
Chik_E2 Forward	5'- GTCACTCATGTACGCACCCA -3'
Chik_E2 Reverse	5'- TATACGGCTCGTTGTTGCCC -3'

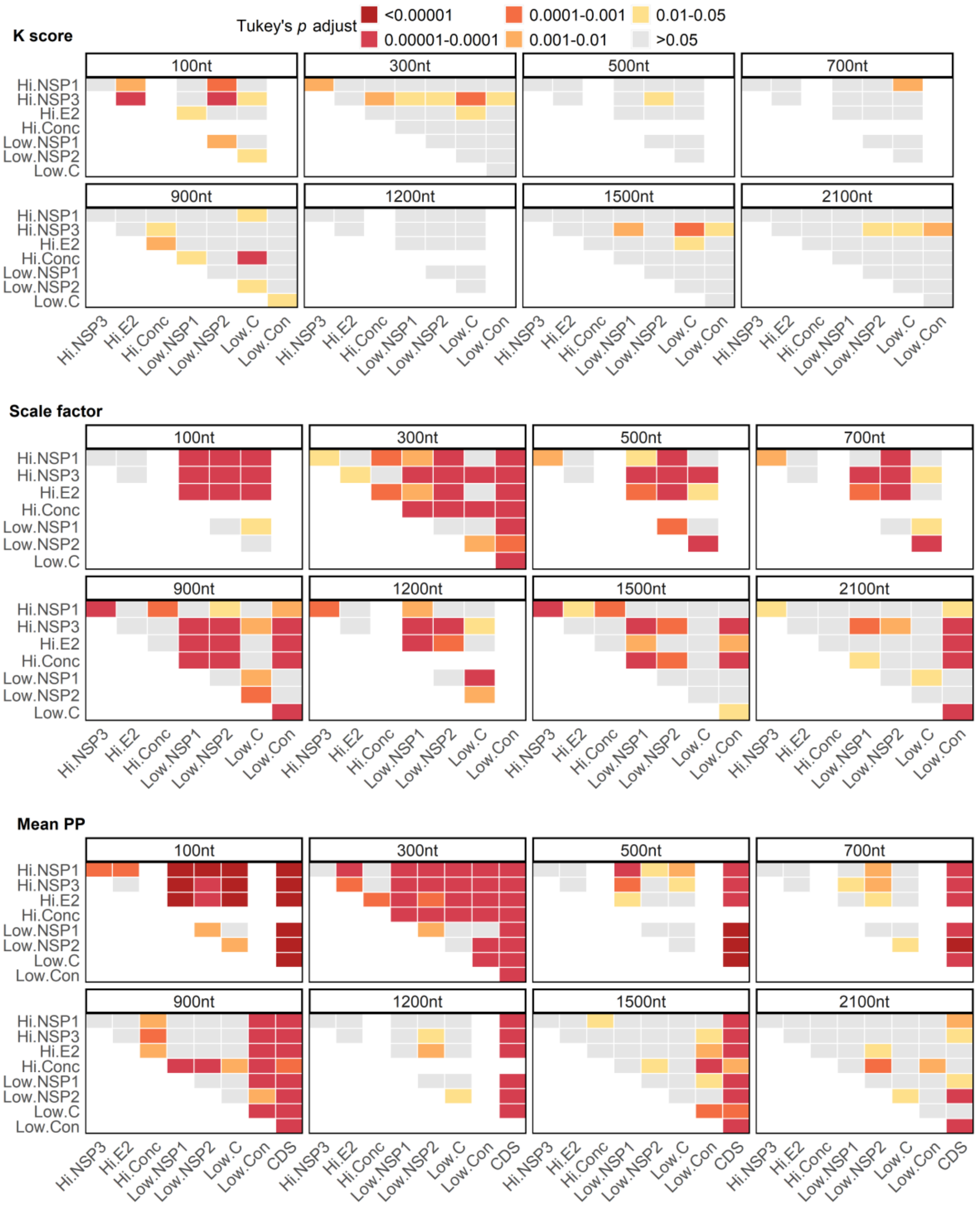

Figure S1. Detailed pairwise statistical comparisons of scaling factors, K-scores, and Mean PP values between all analyzed regions.

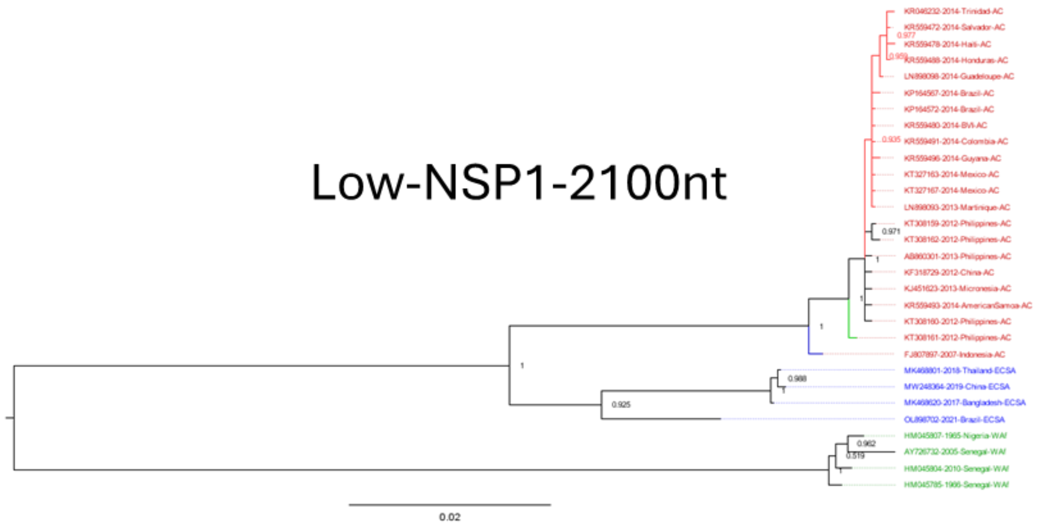
Low-NSP1-2100nt
KR046232-2014-Trinidad-AC
KR559472-2014-Salvador-AC
0.977
KR559478-2014-Haiti-AC
0.959
KR559488-2014-Honduras-AC
LN898098-2014-Guadeloupe-AC
KP164567-2014-Brazil-AC
KP164572-2014-Brazil-AC
KR559480-2014-BVI-AC
0.935
KR559491-2014-Colombia-AC
KR559496-2014-Guyana-AC
KT327163-2014-Mexico-AC
KT327167-2014-Mexico-AC
LN898093-2013-Martinique-AC
KT308159-2012-Philippines-AC
0.971
KT308162-2012-Philippines-AC
AB860301-2013-Philippines-AC
KF318729-2012-China-AC
KJ451623-2013-Micronesia-AC
KR559493-2014-AmericanSamoa-AC
KT308160-2012-Philippines-AC
KT308161-2012-Philippines-AC
FJ807897-2007-Indonesia-AC
MK468801-2018-Thailand-ECSA
0.988
MW248364-2019-China-ECSA
MK468620-2017-Bangladesh-ECSA
0.925
OL898702-2021-Brazil-ECSA
HM045807-1965-Nigeria-WAf
0.962
0.519
AY726732-2005-Senegal-WAf
HM045804-2010-Senegal-WAf
HM045785-1966-Senegal-WAf
0.02

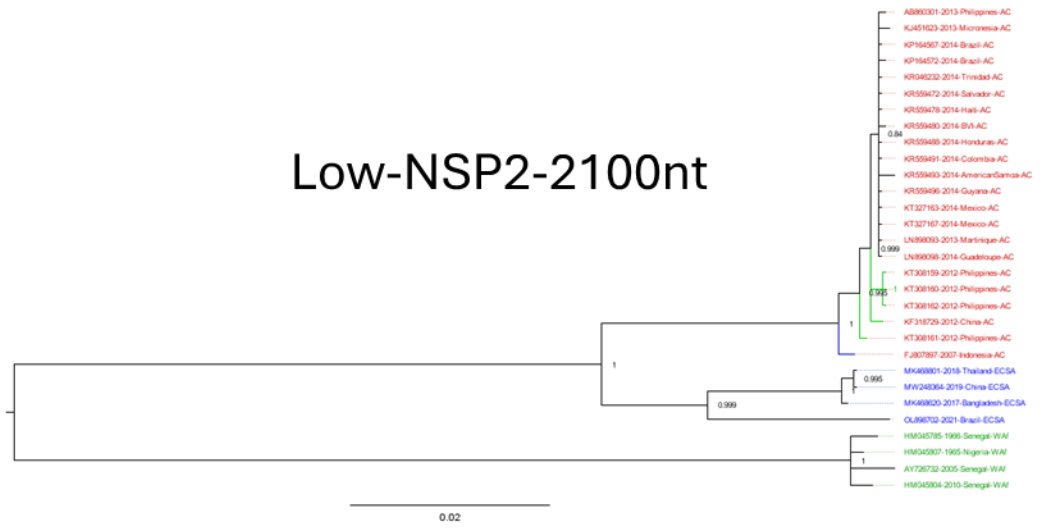
Low-NSP2-2100nt
AB860301-2013-Philippines-AC
KJ451623-2013-Micronesia-AC
KP164567-2014-Brazil-AC
KP164572-2014-Brazil-AC
KR046232-2014-Trinidad-AC
KR559472-2014-Salvador-AC
KR559478-2014-Haiti-AC
KR559480-2014-BVI-AC
0.84
KR559488-2014-Honduras-AC
KR559491-2014-Colombia-AC
KR559493-2014-AmericanSamoa-AC
KR559496-2014-Guyana-AC
KT327163-2014-Mexico-AC
KT327167-2014-Mexico-AC
LN898093-2013-Martinique-AC
0.999
LN898098-2014-Guadeloupe-AC
KT308159-2012-Philippines-AC
KT308160-2012-Philippines-AC
KT308162-2012-Philippines-AC
KF318729-2012-China-AC
KT308161-2012-Philippines-AC
FJ807897-2007-Indonesia-AC
MK468801-2018-Thailand-ECSA
0.995
MW248364-2019-China-ECSA
MK468620-2017-Bangladesh-ECSA
0.999
OL898702-2021-Brazil-ECSA
HM045785-1966-Senegal-WAf
HM045807-1965-Nigeria-WAf
AY726732-2005-Senegal-WAf
HM045804-2010-Senegal-WAf
0.02

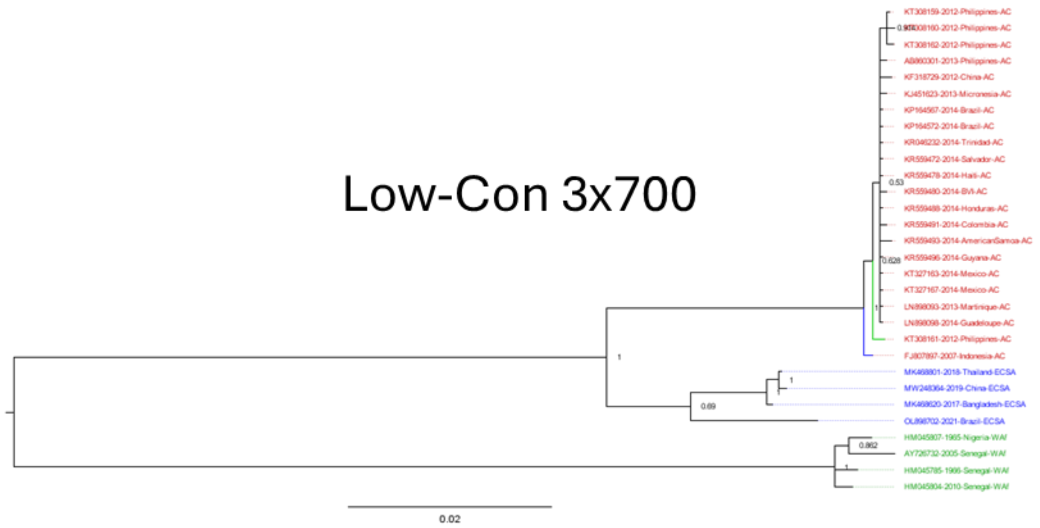
Low-Con 3x700
KT308159-2012-Philippines-AC
KT308160-2012-Philippines-AC
KT308162-2012-Philippines-AC
AB860301-2013-Philippines-AC
KF318729-2012-China-AC
KJ451623-2013-Micronesia-AC
KP164567-2014-Brazil-AC
KP164572-2014-Brazil-AC
KR046232-2014-Trinidad-AC
KR559472-2014-Salvador-AC
KR559478-2014-Haiti-AC
0.53
KR559480-2014-BVI-AC
KR559488-2014-Honduras-AC
KR559491-2014-Colombia-AC
KR559493-2014-AmericanSamoa-AC
0.628
KR559496-2014-Guyana-AC
KT327163-2014-Mexico-AC
KT327167-2014-Mexico-AC
LN898093-2013-Martinique-AC
LN898098-2014-Guadeloupe-AC
KT308161-2012-Philippines-AC
FJ807897-2007-Indonesia-AC
MK468801-2018-Thailand-ECSA
MW248364-2019-China-ECSA
MK468620-2017-Bangladesh-ECSA
0.69
OL898702-2021-Brazil-ECSA
HM045807-1965-Nigeria-WAf
0.862
AY726732-2005-Senegal-WAf
HM045785-1966-Senegal-WAf
HM045804-2010-Senegal-WAf
0.02

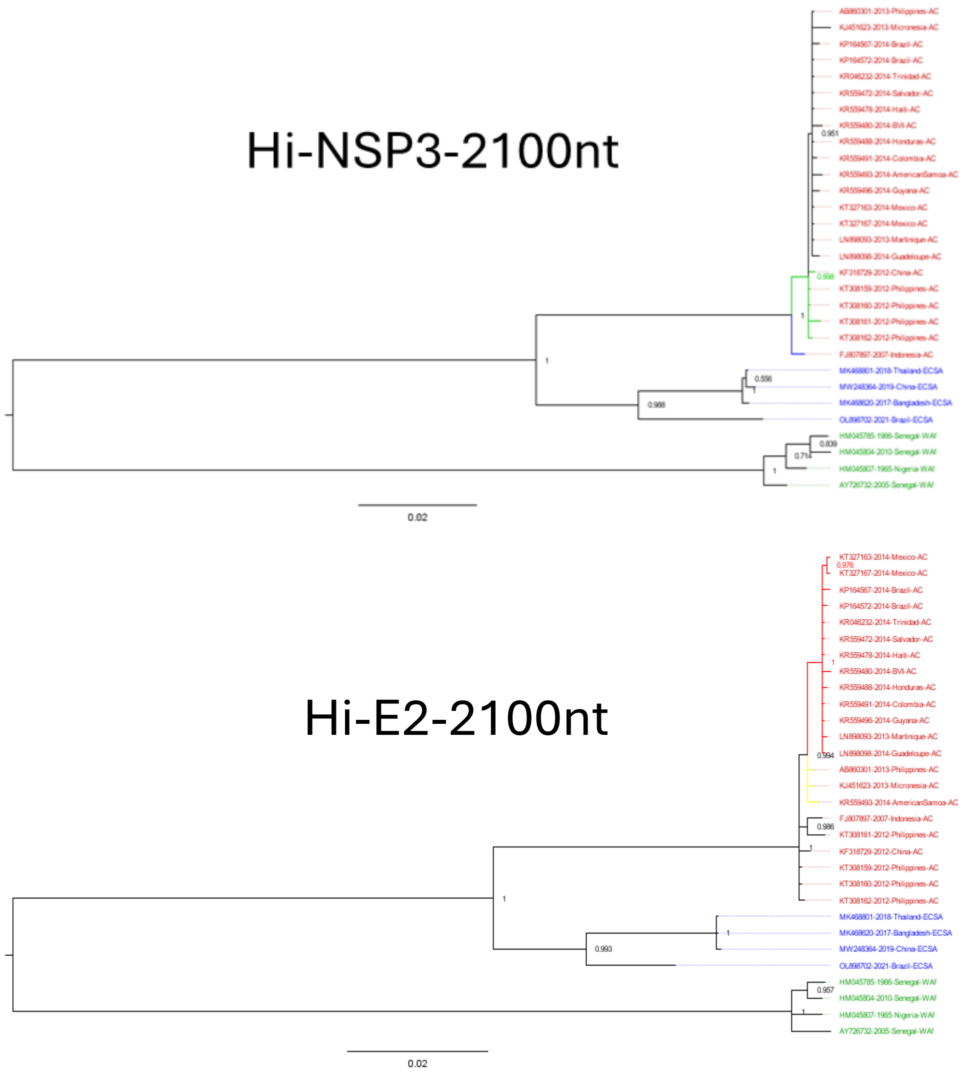

Figure S2: Phylogenetic trees with limited resolution for evolutionary relationships among CHIKV isolates from the American outbreak. The trees constructed with the different regions (Hi- and Low-variables) of 2100 nt length were compared with the one constructed with their respective CDS to assess the congruence between the evolutionary relationships inferred from these distinct genotypes and geographic regions.
